# Burden of untreated transthyretin amyloid cardiomyopathy on patients and their caregivers by disease severity: results from a multicenter, non-interventional, real-world study

**DOI:** 10.3389/fcvm.2023.1238843

**Published:** 2023-08-29

**Authors:** Lucia Ponti, Kristen Hsu, Thibaud Damy, Eduardo Villacorta, Nicolas Verheyen, Denis Keohane, Ronnie Wang, Monica Ines, Nisith Kumar, Carmen Munteanu, Francesco Cappelli

**Affiliations:** ^1^ University of Urbino Carlo Bo, Urbino, Italy; ^2^ Amyloidosis Research Consortium, Newton, MA, United States; ^3^ Referral Center for Cardiac Amyloidosis, CHU Henri Mondor, Créteil, France; ^4^ Complejo Asistencial Universitario de Salamanca, Salamanca, Spain; ^5^ Medical University of Graz, Graz, Austria; ^6^ Pfizer Inc., New York, NY, United States; ^7^ Pfizer Inc., Groton, CT, United States; ^8^ Pfizer Inc., Porto Salvo, Portugal; ^9^ Tuscan Regional Amyloidosis Referral Centre, Careggi University Hospital, Florence, Italy

**Keywords:** caregiver burden, healthcare surveys, heart failure, patient-reported outcome measures, chronic heart failure

## Abstract

**Background:**

The humanistic burden of transthyretin amyloid cardiomyopathy (ATTR-CM) is poorly defined.

**Methods:**

An international study to comprehensively characterize the burden of ATTR-CM on patients naïve to disease-modifying therapy and their unpaid primary caregivers using study-specific and established surveys (patients: Kansas City Cardiomyopathy Questionnaire Overall Summary [KCCQ-OS], 12-Item Short Form Health Survey [SF-12], Hospital Anxiety and Depression Scale [HADS], Patient-Reported Outcomes Measurement Information System [PROMIS] Fatigue and Dyspnea; caregivers: SF-12, HADS, PROMIS Fatigue, Zarit Burden Interview [ZBI]). All data were summarized descriptively.

**Results:**

208 patient and caregiver pairs were included. 86% of patients were male, median age was 81 years, and 91% (141/155 with genetic testing) had wild-type ATTR-CM. Patient responses characterized the mental and physical burden of ATTR-CM, which was numerically higher among those who were New York Heart Association (NYHA) class III (*n* = 43) vs. class I/II (*n* = 156). NYHA class III patients had particularly low KCCQ-OS (36) and SF-12 physical component (27) scores, and 67% had a HADS depression score ≥8. Caregivers (median age 68 years; 85% female; 59% spouse of the patient; median duration of caregiving 1.5 years) reported that NYHA III patients more frequently required help with a range of physical activities than NYHA class I/II patients. 51% of caregivers to NYHA class III patients reported at least a mild-to-moderate burden in the ZBI. A plain language summary of this paper can be found as a supplemental material.

**Conclusions:**

Untreated ATTR-CM is a burden to both patients and their caregivers.

## 1. Introduction

Transthyretin amyloid cardiomyopathy (ATTR-CM) is a condition caused by the deposition of transthyretin amyloid fibrils in the myocardium (1). Wild-type ATTR-CM (ATTRwt-CM) occurs with aging. It typically has a late onset (∼70 years) and a predominantly cardiac phenotype (2). Variant ATTR-CM (ATTRv-CM) has a more variable onset and more commonly presents alongside neurological symptoms (1–4). Although ATTR-CM can have varying presentation, it is consistently associated with reduced lifespan due to progressive heart failure (1, 2). ATTR-CM has historically been underdiagnosed, and poor disease awareness contributes to treatment delays that can negatively impact outcomes (5–8).

The symptoms of ATTR-CM are a burden to patients and caregivers. A previous qualitative study of patients with ATTR-CM and their family members found diverse symptoms reported across multiple physiological systems (in particular, cardiovascular, neurological, and gastrointestinal systems, and mental health) (9). These symptoms required many patients to change their way of life and sacrifice participation in daily activities (9). This study also described the vital role that caregivers and family members play in sharing the disease burden and providing coping mechanisms (9).

Previous studies evaluating the humanistic burden of transthyretin amyloidosis suggest a significant physical, emotional, and social impact on patients and their caregivers (9–20). However, most of these studies focused on patients with variant transthyretin amyloidosis, which can have a predominantly neurological phenotype (10–12, 14, 16, 19). Only a few studies have specifically evaluated the burden of ATTR-CM (9, 13, 15, 17, 20).

This was the first multicenter, international, real-world study aiming to evaluate the burden of ATTR-CM on patients who were naïve to disease-modifying treatment and their unpaid primary caregivers. Findings were used to explore the relationship between burden and ATTR-CM disease severity in both groups. A plain language summary of this study can be found in the Supplemental Materials.

## 2. Methods

### 2.1. Study and participants

This was an international, multicenter, cross-sectional, non-interventional study conducted at amyloidosis centers of excellence and referral centers for patients with ATTR-CM.

Patients with a diagnosis of ATTR-CM (per routine regional practice) and aged 18–89 years were recruited for the study by their doctor (the study investigator). Each patient must have had a predominantly cardiac ATTR amyloidosis phenotype, be naïve to disease-modifying treatment (symptomatic standard of care treatment was permitted), and not have received a heart or liver transplant, left ventricular assist device, or a previous diagnosis of immunoglobulin light chain amyloidosis.

Caregivers must have been aged 18–89 years, unpaid, and the main care provider for the patient with ATTR-CM. They must not have had ATTR-CM themselves or any other disease that significantly impacted their perceived quality of life.

All patients and caregivers provided informed consent to participate in the study. Consent forms were approved by the institutional review board or independent ethics committee at each site before use.

### 2.2. Surveys and analysis

Patients and caregivers completed individual surveys. From each pair, both patient and caregiver must have provided a minimum set of responses to be included in the analysis. The investigator also provided information derived from the patient's available medical records and an interview (in-person or remote).

It was planned for all data to be summarized descriptively. Patient/caregiver-reported outcome measures were additionally examined by the patient's New York Heart Association (NYHA) functional class (21). No statistical tests were conducted to compare NYHA subgroups (I/II and III) as this was not a planned analysis and subgroup characteristics are not well-balanced. Interpretations are based on observed numerical differences.

#### 2.2.1. Surveys self-completed by patients

Surveys self-completed by patients included a range of questions quantifying disease burden on daily life, including symptom severity over the last 7 days (scale of 0–10) and frequency of healthcare visits in the prior 3 months. Established patient-reported outcome surveys completed by patients included the Kansas City Cardiomyopathy Questionnaire (KCCQ), the 12-Item Short Form Health Survey (SF-12) V2, the Hospital Anxiety and Depression Scale (HADS), Patient-Reported Outcomes Measurement Information System (PROMIS) Fatigue Severity Short Form 7a V1.0, and PROMIS Dyspnea Severity Short Form 10a V1.0.

The KCCQ is a 23-item questionnaire assessing how patients perceived the impact of heart failure on their health status in the prior 2 weeks (22, 23). Scores were transformed onto a 0–100 scale, with higher scores indicating better health status. The total symptoms score is the mean of the symptom domain scores, excluding symptom stability. The clinical summary score is the mean of the total symptoms and physical limitation scores. The overall summary (KCCQ-OS) score is the mean of total symptoms, physical limitation, social limitations, and quality of life scores. Previously published interpretations of KCCQ-OS scores were used: 0–24 denotes very poor to poor; 25–49, poor to fair; 50–74, fair to good; and 75–100, good to excellent health (22).

The SF-12 assesses health status in the prior 7 days (24, 25). Scores were calculated using the US algorithm and range from 0 to 100, with higher scores representing better self-reported health. The US general population median for the physical component is 54 (25th percentile, 47, 75th percentile, 56) and for the mental component is 53 (45, 57) (26). Reference scores for the US population have a high degree of correspondence with European populations and are often used as the basis for interpretation (24, 26, 27). Reference scores differ by age, and in particular, the physical component is known to significantly decline with aging (26, 28). Among the US general population aged ≥75 years, the median physical component score is 39 (29, 48) and the median mental component is 54 (40, 59) (26). These values are broadly similar to averages calculated in European general populations aged ≥65 years (28–30).

The HADS is a 14-item questionnaire with anxiety and depression subscales ranging from 0 to 21 (31). Higher scores indicate more severe anxiety and depression. Scores ≥8 were considered to represent potentially clinically relevant anxiety or depression (32, 33).

The PROMIS Fatigue Short Form is a 7-item questionnaire assessing the severity of daily fatigue over the past 7 days (34). The PROMIS Dyspnea Short Form is a 10-item questionnaire assessing the severity of dyspnea over the past 7 days (35). In both, patients provided responses on a 5-point Likert scale (1 = never; 2 = rarely; 3 = sometimes; 4 = often; 5 = always). A T-score was produced according to PROMIS Fatigue scoring manual (dated April 28, 2021) or PROMIS Dyspnea scoring manual (dated May 17, 2021). For PROMIS Fatigue, the mean T-score for the US population is 50 with a standard deviation of 10 (34). This has been shown to be similar in the Dutch general population (36). Higher scores indicate more severe fatigue. For PROMIS Dyspnea, the mean T-score for patients with chronic obstructive pulmonary disease is 50 with a standard deviation of 10. Higher scores indicate more severe dyspnea (37).

#### 2.2.2. Surveys self-completed by caregivers

Surveys self-completed by caregivers included a range of questions quantifying the burden of care on daily life and the frequency of assistance provided to the patient for attendance of healthcare visits in the prior 3 months. The HADS, SF-12, and PROMIS Fatigue assessments were completed and scored as described for patients. Caregivers also completed the 22-item Zarit Burden Interview (ZBI) (38, 39). Questions were answered on a 5-point Likert scale (0 = never; 1 = rarely; 2 = sometimes; 3 = quite frequently; 4 = nearly always). The total score ranges from 0 to 88, with a higher score indicating a higher level of burden. Burden levels were defined as: 0–20, little or no burden; 21–40, mild to moderate burden; 41–60, moderate to severe burden; and 61–88, severe burden.

#### 2.2.3. Data provided by the investigator

The investigator provided information on the patient's age, sex, underlying conditions, comorbidities, *TTR* genotype, time from diagnosis, current symptoms, NYHA class, and left ventricular ejection fraction (LVEF). They also provided information on the caregiver's age, sex, and underlying conditions.

## 3. Results

A plain language summary of this paper can be found in the Supplemental Materials.

From July 2021 to August 2022, 208 patient and caregiver pairs were enrolled and self-completed the assessments. Of these, 95 pairs were from Italy, 34 from France, 31 from Spain, 17 from Australia, 15 from Austria, 10 from Canada, and 6 from Russia. Another 10 pairs were initially enrolled but did not complete the minimum set of responses needed to be included in the analysis.

### 3.1. Demographics and clinical characteristics of untreated patients with ATTR-CM

The patient population had a median age of 81.0 years and 86.1% were male (Table 1). The majority were married or in a domestic partnership (80.3%) and retired (95.2%). Overall, patients were relatively newly diagnosed (median 0.5 years ago) and reported ∼1.5 years from first symptoms to diagnosis. Diagnoses were made per routine regional practices, and a range of diagnostic methods had been used, with 91.8% of diagnoses involving scintigraphy and 82.7% other imaging modalities. Diagnostic biopsies were taken in just under one-third (29.3%) of patients. Genetic testing results were not available for 53 patients at the time of the study (majority pending), but of the 155 patients with data, 91.0% had ATTRwt-CM. Comorbidities, as reported by the investigator, were diverse among the patient population, most commonly including heart failure and hypertension. At the time of the survey, 10.6% of patients had diagnosed comorbid depression, and 8.2% comorbid anxiety (Table 2). Not all patients had an NYHA classification recorded. Among the 199 patients with a classification, nearly two-thirds (60.3%) were NYHA class II. Similar proportions of patients were class I (18.1%) or III (21.6%), and none were class IV. In total, 197 patients had an LVEF recorded. Of these, the majority had a normal LVEF (≥55% in 52.3%); only 8 (4.1%) had an LVEF <35% (severely below normal).

**Table 1:** Physician-reported patient demographics and clinical characteristics by NYHA class.

	All patients *N* = 208	Patients who were NYHA class I/II *n* = 156	Patients who were NYHA class III *n* = 43
Age			
Mean (SD)	79.6 (7.0)	79.4 (6.8)	79.8 (8.0)
Median (min, max)	81.0 (46, 90)	81.0 (46, 90)	81.0 (47, 90)
Sex, *n* (%)			
Male	179 (86.1)	136 (87.2)	34 (79.1)
Female	29 (13.9)	20 (12.8)	9 (20.9)
Disease duration, years (IQR)			
Age at first symptoms, median	78.5 (74.0, 82.0)	78.0 (74.0, 82.0)	78.0 (73.0, 83.0)
Age at diagnosis, median	80.0 (75.0, 84.0)^a^	80.0 (75.0, 83.0)^b^	79.0 (75.0, 85.0)
Years since diagnosis, median	0.5 (0.1, 1.3)^a^	0.5 (0.1, 1.3)^b^	0.3 (0.1, 1.2)
Diagnostic method, n* (%)			
Scintigraphy	191 (91.8)	144 (92.3)	38 (88.4)
Other imaging	172 (82.7)	133 (85.3)	30 (69.8)
Symptoms	131 (63.0)	100 (64.1)	26 (60.5)
Genetic testing	107 (51.4)	81 (51.9)	23 (53.5)
Physical examination	80 (38.5)	58 (37.2)	19 (44.2)
Biopsy	61 (29.3)	44 (28.2)	17 (39.5)
Heart	24 (39.3)	15 (34.1)	9 (52.9)
Salivary gland	28 (45.9)	23 (52.3)	5 (29.4)
Fat pad	6 (9.8)	5 (11.4)	1 (5.9)
Carpal ligament	3 (4.9)	1 (2.3)	2 (11.8)
Family history	3 (1.4)	3 (1.9)	0
Type of ATTR-CM, *n* (%)			
*n* with genetic testing	155	119	29
Wild-type	141 (91.0)	109 (91.6)	25 (86.2)
Variant^c^	14 (9.0)	10 (8.4)	4 (13.8)
NYHA class, *n* (%)			
*n* with classification	199	156	43
I	36 (18.1)	36 (23.1)	0
II	120 (60.3)	120 (76.9)	0
III	43 (21.6)	0	43 (100.0)
IV	0	0	0
Left ventricular ejection fraction, *n* (%)			
*n* with measure	197	149	42
≥55%	103 (52.3)	80 (53.7)	20 (47.6)
40%–54%	77 (39.1)	56 (37.6)	18 (42.9)
≤39%	17 (8.6)	13 (8.7)	4 (9.5)

ATTR-CM, transthyretin amyloid cardiomyopathy; IQR, interquartile range; NYHA, New York Heart Association; SD, standard deviation.

^a^ *n* = 207.

^b^ *n* = 155.

^c^ Including *n* = 7 with *I68l* (*p.I88l;* 1 NYHA class III), 1 with *V30M* (*p.V50M;* NYHA class III), 1 with *V122I* (*p.V142I;* NYHA class III), and 4 with other transthyretin gene variations (1 NYHA class III).

* More than one can apply.

**Table 2:** Physician-reported patient comorbidities and current therapies by NYHA class.

	All patients *N* = 208	Patients who were NYHA class I/II *n* = 156	Patients who were NYHA class III *n* = 43
Comorbidities, *n* (%)*			
Heart failure	115 (55.3)	86 (55.1)	29 (67.4)
Hypertension	115 (55.3)	80 (51.3)	29 (67.4)
Heart failure with preserved ejection fraction	100 (48.1)	78 (50.0)	20 (46.5)
Carpel tunnel syndrome	100 (48.1)	78 (50.0)	20 (46.5)
Hyperlipidemia	84 (40.4)	53 (34.0)	23 (53.5)
Diabetes mellitus	39 (18.8)	28 (17.9)	9 (20.9)
Kidney disease	36 (17.3)	20 (12.8)	12 (27.9)
Cancer (other than skin)	32 (15.4)	19 (12.2)	11 (25.6)
Neuropathy	24 (11.5)	14 (9.0)	10 (23.3)
Depression	22 (10.6)	16 (10.3)	5 (11.6)
Obesity	21 (10.1)	13 (8.3)	6 (14.0)
Anxiety	17 (8.2)	12 (7.7)	4 (9.3)
Liver disease	5 (2.4)	4 (2.6)	1 (2.3)
Autonomic nervous system disease	4 (1.9)	2 (1.3)	2 (4.7)
Other^†^	109 (52.4)	78 (50.0)	24 (55.8)
Current symptomatic therapies, *n* (%)*			
Anti-hypertensives	174 (83.7)	126 (80.8)	39 (90.7)
Diuretics	145 (69.7)	105 (67.3)	36 (83.7)
Anticoagulants	132 (63.5)	100 (64.1)	23 (53.5)
Antiarrhythmic therapy including implanted cardioverter-defibrillators	65 (31.3)	58 (37.2)	6 (14.0)
Antidepressants	24 (11.5)	17 (10.9)	7 (16.3)
Steroids	17 (8.2)	15 (9.6)	1 (2.3)
Drugs for neuropathy	14 (6.7)	10 (6.4)	4 (9.3)

NYHA, New York Heart Association.

* More than one can apply.

^†^ “Other” was a pre-specified category selected by the investigator. The nature of the comorbidity was not indicated.

Aside from age, the demographic, diagnostic, and clinical characteristics of untreated patients with ATTR-CM who were NYHA class I/II or III were generally similar (Tables 1, 2). Among patients with genetic testing data, a slightly higher proportion of patients who were NYHA class III had ATTRv-CM than in patients who were NYHA class I/II (13.8% vs. 8.4%, respectively). Comorbidities were present in comparable or numerically higher proportions of patients who were NYHA class III than in patients who were NYHA class I/II. LVEF was poorest in patients who were NYHA class III.

### 3.2. Burden of untreated ATTR-CM to patients by disease severity

**Figure 1:**
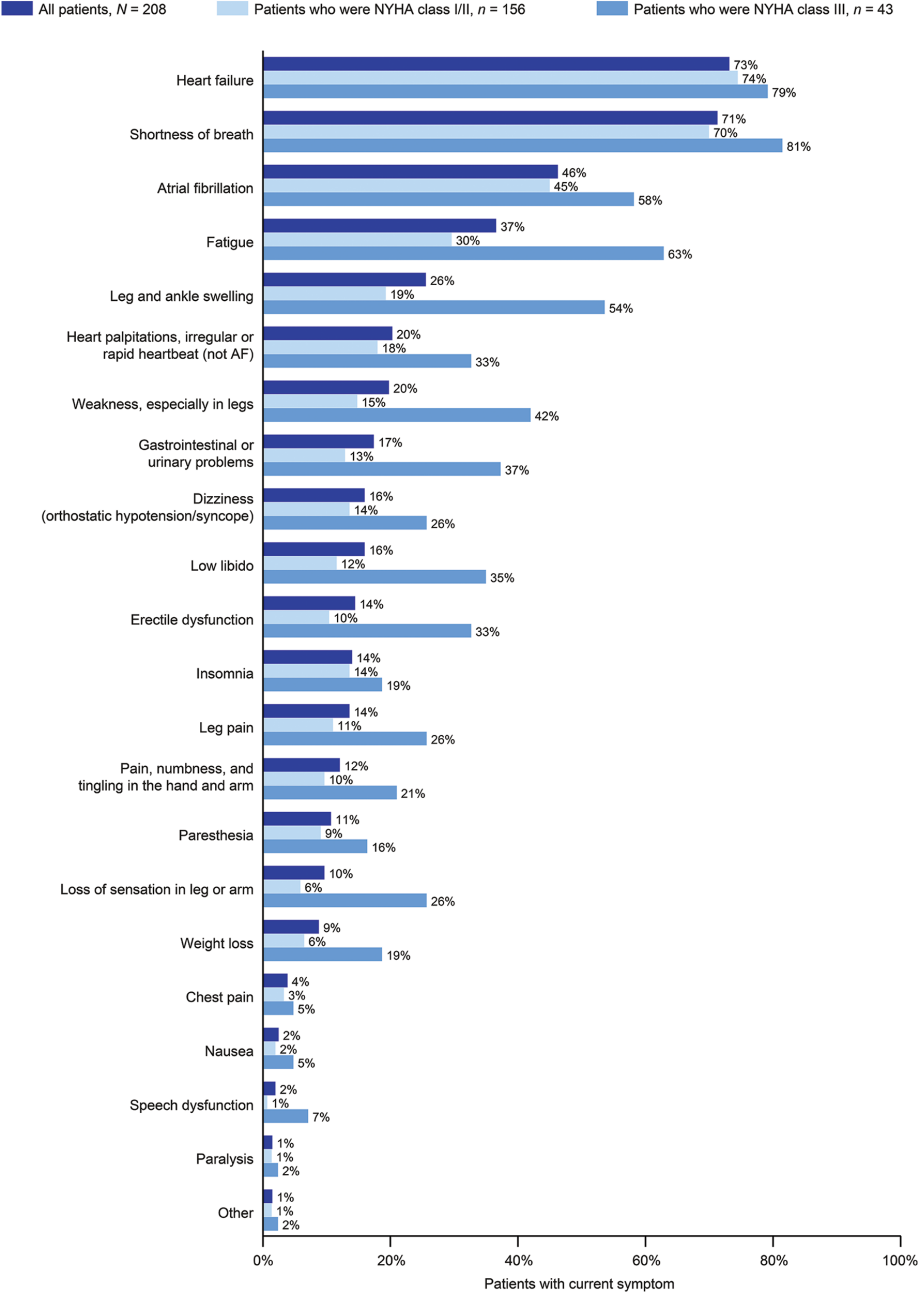
Current symptoms in patients with ATTR-CM by NYHA class. 199/208 patients had NYHA classification data. AF, atrial fibrillation; ATTR-CM, transthyretin amyloid cardiomyopathy; NYHA, New York Heart Association.

Physicians reported on the ATTR-CM symptoms currently experienced by patients from a pre-defined list (Figure 1). Current symptoms derived from multiple physiological systems but the most common among all patients were heart failure (73.1%) and shortness of breath (71.2%). More than a quarter of all patients were currently experiencing atrial fibrillation (46.2%), fatigue (36.5%), or leg and ankle swelling (25.5%). Neurological symptoms were also common, including dizziness (15.9%), erectile dysfunction (14.4%), leg pain (13.5%), pain, numbness and tingling in the hand or arms (12.0%), paresthesia (10.6%), and loss of sensation in the limbs (9.6%). All symptoms were experienced in numerically higher proportions of patients who were NYHA class III than in patients who were NYHA class I/II.

Current symptomatic therapies received by ≥50% of all patients included anti-hypertensives (83.7%), diuretics (69.7%), and anticoagulants (63.5%; Table 2). There were some numerical differences in the proportion of patients currently using each type of symptomatic therapy, including a higher proportion of patients who were NYHA class III vs. I/II using diuretics, anti-hypertensives, antidepressants, and drugs for neuropathy. Conversely, higher proportions of patients who were NYHA class I/II vs. III currently took antiarrhythmic therapy, anticoagulants, and steroids.

**Figure 2:**
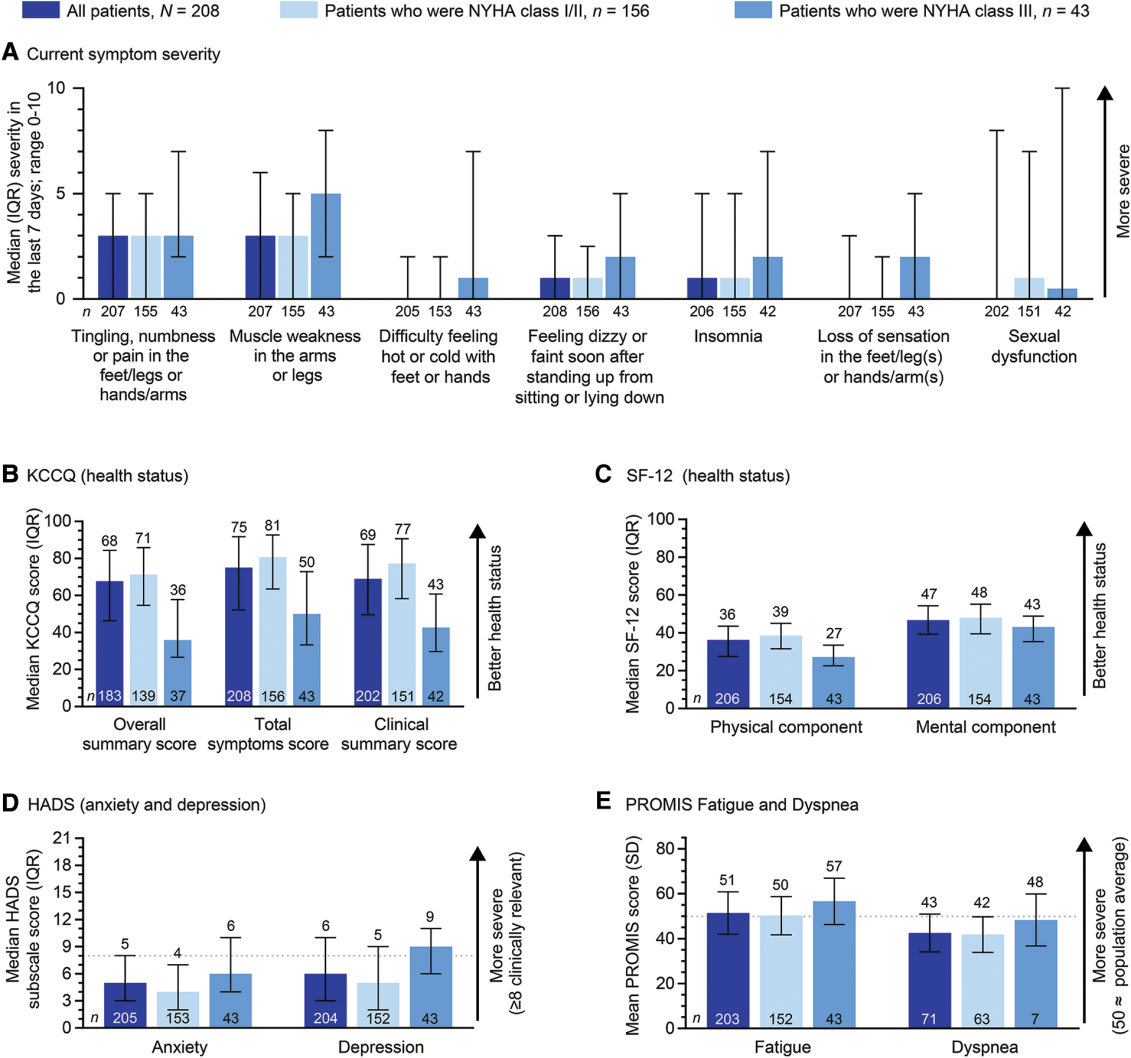
Patient-reported ATTR-CM burden by NYHA class. 199/208 patients had NYHA classification data. In (**A**), symptoms with a severity of one or more in any group are shown. ATTR-CM, transthyretin amyloid cardiomyopathy; HADS, Hospital Anxiety and Depression Scale; IQR, interquartile range; KCCQ, Kansas City Cardiomyopathy Questionnaire; NYHA, New York Heart Association; PROMIS, Patient-Reported Outcome Measurement Information System; SD, standard deviation; SF-12, 12-item Short Form Health Survey.

Patients with ATTR-CM self-reported significant physical burdens, with more than half of patients (61.7%) being unable to walk normally and just less than one-third unable to participate in social and household activities (Table 3). Being unable to walk normally or participate in social or household activities were numerically more common in patients who were NYHA class III than in patients who were NYHA class I/II. When patients were asked about the severity of specific symptoms, those of a neurological nature were most bothersome, with tingling, numbness, or pain in the limbs and muscle weakness in the arms or legs both being rated 3/10 for severity in the prior 7 days (Figure 2A).

**Table 3:** Patient-reported physical burden of ATTR-CM by NYHA class.

	All patients *N* = 208	Patients who were NYHA class I/II *n* = 156	Patients who were NYHA class III *n* = 43
Unable to walk normally, *n* (%)	127 (61.7%) [*n* = 206]	86 (55.8%) [*n* = 154]	36 (83.7%) [*n* = 43]
Unable to participate in social or leisure activities in the past 3 months, *n* (%)	59 (28.9) [*n* =^ ^204]	36 (23.7) [*n* = 152]	23 (53.5) [*n* = 43]
Median days (IQR)	25.0 (10.0, 85.0) [*n* = 49]	11.0 (5.0, 80.0) [*n* = 30]	80.0 (43.0, 90.0) [*n* = 19]
Unable to do household chores in the past 3 months, *n* (%)	55 (27.1) [*n* = 203]	31 (20.5) [*n* = 151]	22 (51.2) [*n* = 43]
Median days (IQR)	48.0 (10.0, 90.0) [*n* = 46]	20.0 (10.0, 90.0) [*n* = 27]	62.0 (30.0, 92.0) [*n* = 19]
Family member had to do your household chores because of your ATTR-CM in the past 3 months, *n* (%)	63 (31.0) [*n* = 203]	34 (22.4) [*n* = 152]	26 (61.9) [*n* = 42]
Median days (IQR)	62.0 (10.0, 90.0) [*n* = 51]	66.0 (10.0, 90.0) [*n* = 28]	62.0 (10.0, 92.0) [*n* = 23]
Hours of chores, median (IQR)	3.0 (2.0, 6.0) [*n* = 52]	3.0 (2.0, 5.0) [*n* = 30]	4.5 (2.0, 12.0) [*n* = 22]

ATTR-CM, transthyretin amyloid cardiomyopathy; IQR, interquartile range; NYHA, New York Heart Association.

With regard to healthcare utilization, 79.2% of patients (*n* = 207) had attended an outpatient visit in the prior 3 months. Outpatient visits were numerically more common among patients who were NYHA class III vs. patients who were class I/II (93.0% [*n* = 43] vs. 76.1% [*n* = 155]); with similar trends in patient hospitalizations (27.8% overall [*n* = 205]; 41.9% [*n* = 43] vs. 24.8% [*n* = 153]), and emergency room or urgent care visits for ATTR-CM (20.7% overall [*n* = 203]; 32.6% [*n* = 43] vs. 17.9% [*n* = 151]).

Established patient-reported outcome surveys were used to further characterize the burden of untreated ATTR-CM (Figures 2B–E). All scores indicated incrementally higher burden in patients who were NYHA class III as compared with patients who were NYHA class I/II. Median KCCQ-OS, total symptom, and clinical summary scores among all patients were 68 (indicating fair to good health status), 75, and 69, respectively. Patients who were NYHA class III had numerically lower scores vs. patients who were NYHA class I/II (KCCQ-OS 36 vs. 71; total symptom score: 50 vs. 81; and clinical summary score: 43 vs. 77). The KCCQ-OS score indicated poor to fair health status in patients who were NYHA class III, and fair to good health in patients who were NYHA class I/II (22).

Median SF-12 physical and mental component summary scores among all patients were 36 and 47, respectively. These were slightly below published norms for the general population aged ≥75 years (39 and 54) (26). Both physical and mental component summary scores were numerically lower in patients who were NYHA class III than in patients who were NYHA class I/II (27 vs. 39 and 43 vs. 48).

In the HADS depression and anxiety subscales, patients had median scores of 6 and 5, respectively. Medians were numerically higher in patients who were NYHA class III (9 and 6), as compared with patients who were NYHA class I/II (5 and 4). Among patients who were NYHA class III, two-thirds (67%; 29/43) had clinically relevant depression (score ≥8), as compared with one-third (32%; 49/152) of patients who were NYHA class I/II.

The mean PROMIS Fatigue score among all patients was 51, with a slightly higher score in patients who were NYHA class III (57), as compared with those who were NYHA class I/II (50). The PROMIS Fatigue has a general population norm of 50 (SD 10).

The PROMIS Dyspnea questionnaire was completed by less than half of all patients (*n* = 71), with a mean score of 43. There was a slightly higher mean score in patients who were NYHA class III (48), as compared with those who were NYHA class I/II (42). A score of 48 in patients who were NYHA class III suggests similar dyspnea to that experienced by patients with chronic obstructive pulmonary disease [population norm of 50 (SD of 10)].

### 3.3. Demographics of primary caregivers to patients with ATTR-CM

Caregivers had a median age of 68.0 years. The majority were female (84.6%) and a spouse (58.7%) or adult child (37.0%) of the patient (Table 4). Caregivers and patients commonly lived in the same household (66.3% of all caregivers). The demographics among caregivers to patients who were NYHA class I/II and III were broadly similar.

**Table 4:** Caregiver demographics and characteristics by the patient's NYHA class.

	All caregivers *N* = 208	Caregivers to patients who were NYHA class I/II *n* = 156	Caregivers to patients who were NYHA class III *n* = 43
Age			
Mean (SD)	65.8 (12.2)	66.4 (12.6)	65.9 (10.5)
Median (min, max)	68.0 (32, 88)	68.5 (32, 88)	68.0 (45, 83)
Sex, *n* (%)			
Male	32 (15.4)	25 (16.0)	4 (9.3)
Female	176 (84.6)	131 (84.0)	39 (90.7)
Relationship to the patient, *n* (%)			
Spouse	122 (58.7)	95 (60.9)	26 (60.5)
Adult child	77 (37.0)	56 (35.9)	14 (32.6)
Parent	2 (1.0)	1 (0.6)	1 (2.3)
Sibling	1 (0.5)	1 (0.6)	0
Other	6 (2.9)	3 (1.9)	2 (4.7)
Caregiving characteristics			
Caregiver and patient live in the same house, *n* (%)	138 (66.3)	102 (65.4)	34 (79.1)
Hours spent providing care per week, median (IQR)	4.5 (0.0, 27.0) [*n* = 176]	2.0 (0.0, 21.0) [*n* = 130]	17.5 (4.0, 75.0) [*n* = 38]
Years caregiving to date, median (IQR)	1.5 (0.0, 3.0) [*n* = 166]	1.0 (0.0, 3.0) [*n* = 123]	2.0 (1.3, 3.5) [*n* = 37]
Caregivers reporting that there were days in the last 3 months when they were unable to complete typical household chores due to caregiving responsibilities, *n* (%)	21 (10.3) [*n* = 204]	14 (9.2) [*n* = 153]	6 (14.0) [*n* = 43]
Median days (IQR)	7.0 (2.0, 21.0) [*n* = 15]	6.0 (2.0, 20.0) [*n* = 10]	7.0 (4.0, 30.0) [*n* = 5]
Caregivers reporting that there were days in the last 3 months when a family member had to do their household chores due to caregiving responsibilities, *n* (%)	23 (11.4) [*n* = 202]	15 (9.9) [*n* = 152]	7 (16.7) [*n* = 42]
Median days (IQR)	11.0 (3.5, 17.5) [*n* = 20]	11.0 (3.0, 15.0) [*n* = 14]	11.0 (4.0, 20.0) [*n* = 6]

IQR, interquartile range; NYHA, New York Heart Association; SD, standard deviation.

### 3.4. Burden of ATTR-CM on caregivers by patient's disease severity

Caregivers reported that they spent a median of 4.5 h a week providing care to the patient with ATTR-CM. This was over 8-fold higher (17.5 h) among those who cared for patients who were NYHA class III as compared with those who cared for patients who were NYHA class I/II (2.0 h; Table 4). Most had not been caregivers for an extended period, with a median of 1.5 years' duration (2 years to NYHA class III patients; 1 year to NYHA class I/II patients). This duration was longer than the average time since diagnosis for patients but was consistent or less than the average time since first symptoms.

Around 10% of caregivers reported that their caregiving responsibilities meant that they could not complete their typical daily chores at least once in the prior 3 months (median number of days was 7.0; Table 4). This proportion was 14% among caregivers to patients who were NYHA class III and 9% among caregivers to patients who were NYHA class I/II. Similar but slightly higher proportions of caregivers reported that they had to ask another family member to help with their daily chores because of their caregiving responsibilities.

Despite being surveyed during the COVID-19 pandemic, 68.1% of all caregivers (*n* = 207) had accompanied the patient on an outpatient visit in the prior 3 months. This was numerically more common among caregivers to patients who were NYHA class III than among caregivers to patients who were NYHA class I/II (76.7% [*n* = 43] vs. 67.1% [*n* = 155]). Similarly, accompanying the patient to emergency room or urgent care visits was numerically more common among caregivers to patients who were NYHA class III than among caregivers to patients who were NYHA class I/II (16.7% overall [*n* = 204]; 30.2% [*n* = 43] vs. 13.1% [*n* = 153]).

**Figure 3:**
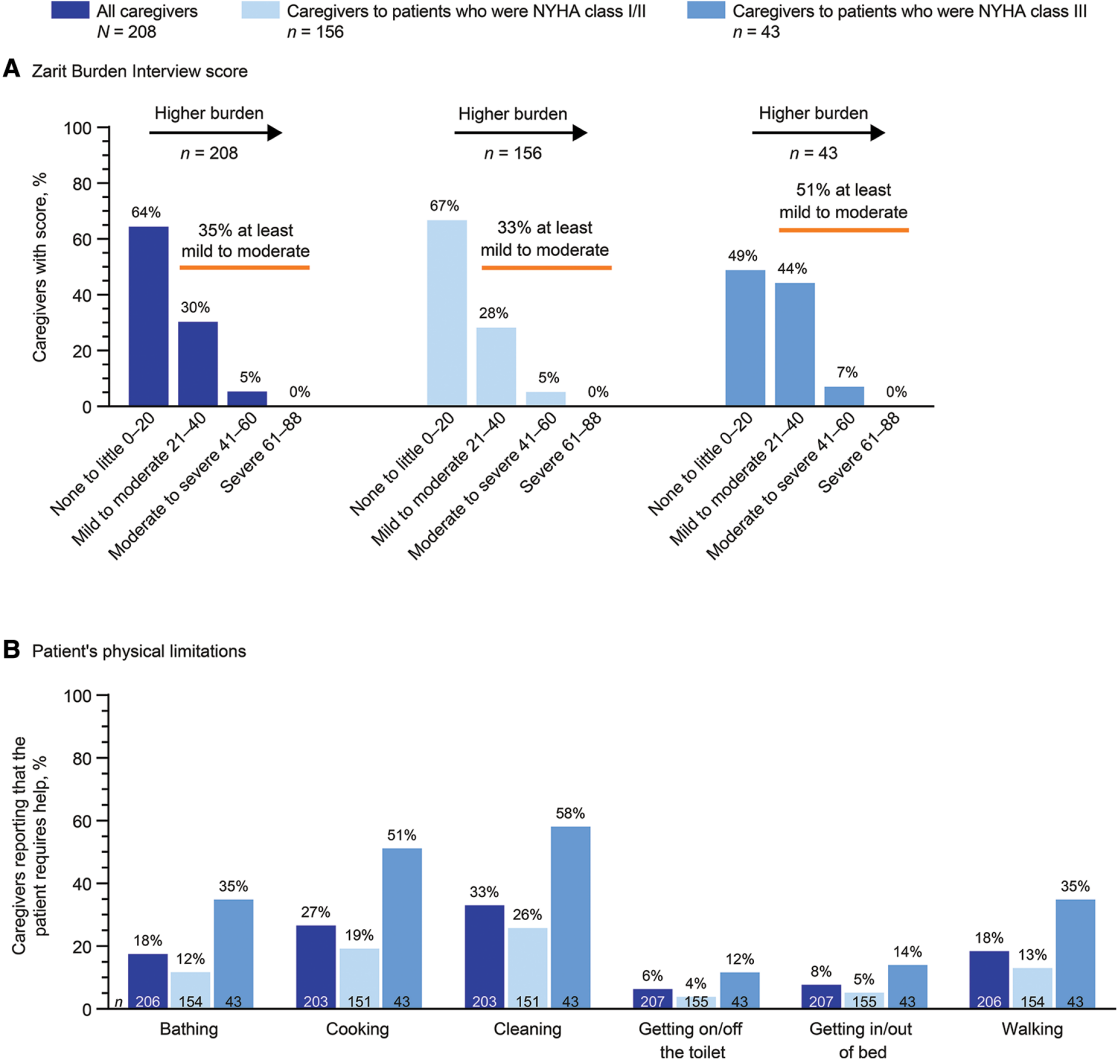
Caregiver-reported burden by the patient's NYHA class. 199/208 patients had NYHA classification data. NYHA, New York Heart Association.

In the ZBI, 35% of all caregivers reported at least a mild to moderate burden of care (score ≥21; Figure 3A). More than half (51%) of caregivers to patients who were NYHA class III reported at least a mild to moderate burden of care, compared with 33% of caregivers to patients who were NYHA class I/II. No caregivers reported a severe burden of care (score ≥61). Consistent with these findings, the median ZBI score was 13 in all caregivers, 14 in caregivers to patients who were NYHA class I/II, and 21 in caregivers to patients who were NYHA class III.

Caregivers reported that patients required help with many everyday physical tasks (Figure 3B). The proportion of patients requiring help with each task was numerically higher among those who were NYHA class III vs. those who were NYHA class I/II, including cleaning (58.1% vs. 25.8%), cooking (51.2% vs. 19.2%), walking (34.9% vs. 13.0%), bathing (34.9% vs. 11.7%), getting in or out of bed (14.0% vs. 5.2%), and getting on or off the toilet (11.6% vs. 3.9%). Caregivers (*n* = 202) reported that 11.4% of patients had some form of incontinence. This proportion was 27.9% in patients who were NYHA class III and 6.6% in patients who were NYHA class I/II.

**Figure 4:**
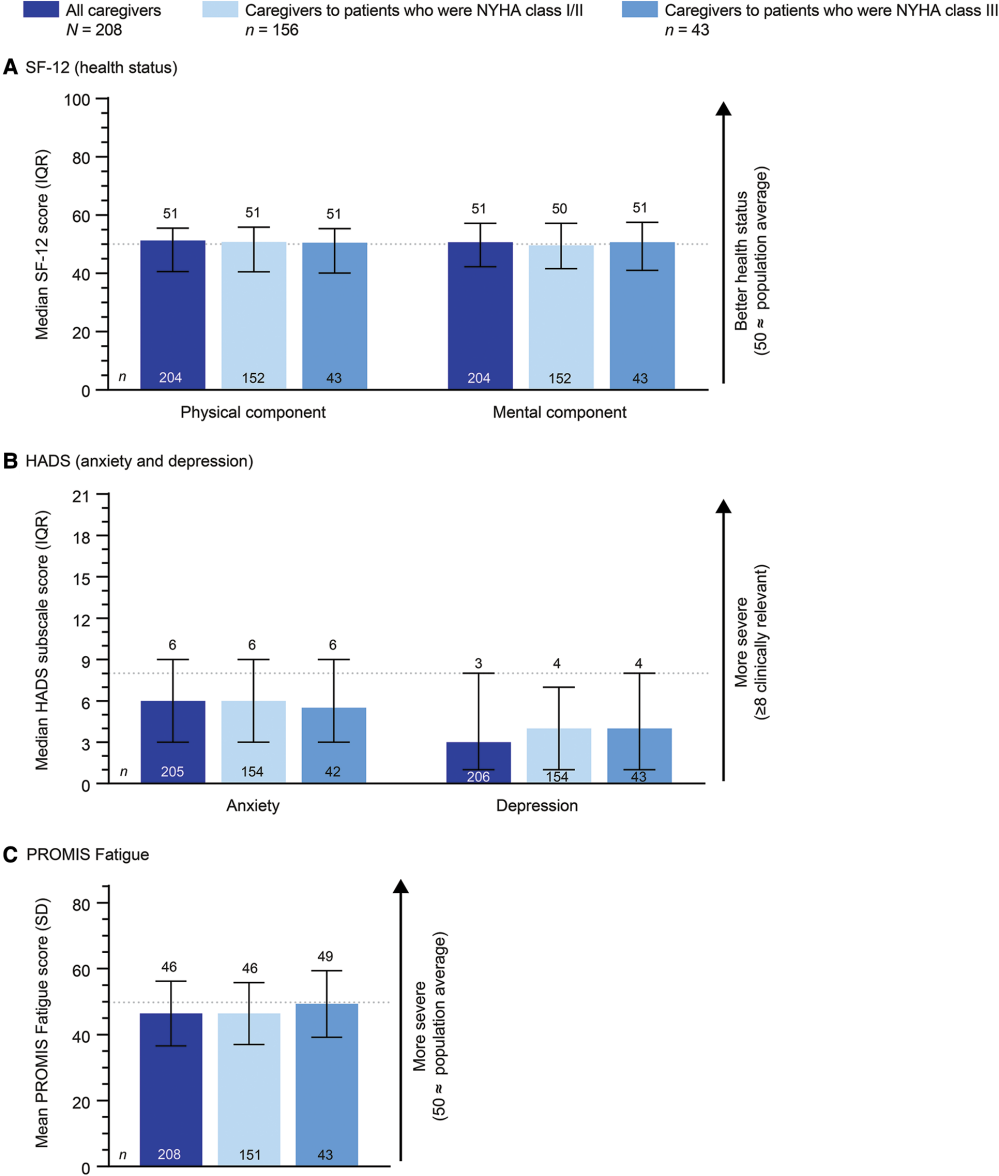
Caregiver-reported scales by the patient's NYHA class. 199/208 patients had NYHA classification data. HADS, Hospital Anxiety and Depression Scale; IQR, interquartile range; NYHA, New York Heart Association; PROMIS, Patient-Reported Outcome Measurement Information System; SD, standard deviation; SF-12, 12-item Short Form Health Survey.

The SF-12, HADS, and PROMIS Fatigue scales showed no meaningful differences in health status, mental health, or fatigue levels reported by caregivers to patients who were NYHA class I/II or III (Figure 4).

## 4. Discussion

This was the first multicenter, international, real-world study to comprehensively characterize the burden of ATTR-CM in patients naïve to disease-modifying therapy and their caregivers. Findings showed that both patients and caregivers felt a mental and physical burden of untreated ATTR-CM, and this burden appeared to increase with disease severity.

Nearly all published assessments of transthyretin amyloidosis burden have evaluated patients with variant transthyretin amyloidosis, which can have a predominantly neurological phenotype (9–20). Although genetic testing data were not available for ∼25% of patients in our study, 91% of those with data were confirmed to have ATTRwt-CM. Assuming this is reflective of those without genetic testing, this suggests that our study population predominantly comprised those with relatively newly diagnosed ATTRwt-CM who were naïve to disease-modifying therapy but receiving standard-of-care symptomatic therapies. Although the reason for the lack of disease-modifying treatment was not captured, it likely reflected local approval or reimbursement status at the time. Findings from our study should be interpreted in the context of the specific population evaluated and should not be extrapolated to patients with variant transthyretin amyloidosis and predominantly neurological phenotype, other forms of amyloidosis, or where patients are receiving disease-modifying treatment. Furthermore, our patient population was generally elderly, male, and experienced a burden that was heavily related to their experiences with physical symptoms. This makes the burden characterized in this study difficult to compare with that arising from conditions affecting younger patients or with a predominantly mental burden (such as dementia). Published burden measures are variable even within specific conditions, further suggesting the sensitivity of disease burden to contextual and study design differences. A particular strength of this study is in the comprehensive and unique characterization of the burden of untreated ATTR-CM in pairs of patients and their primary caregivers. This pairing allows direct association between demographics and characteristics of caregivers and patients.

### 4.1. Burden of ATTR-CM in patients

We found ATTR-CM symptoms to be common and diverse among patients, affecting their ability to do everyday tasks and preventing 62% of patients from walking normally. These symptoms contributed to around one-third of patients reporting inability to participate in social and household activities in the 3 months prior. Similar frustrations around the loss of ability to complete everyday physical tasks were previously reported in a qualitative study of patients with ATTR-CM and their caregivers, where intolerance to activity, inability to exercise, insomnia, and fatigue were the most challenging symptoms of ATTR-CM for patients (9). Although cardiovascular symptoms were most common in our study, neurological symptoms were also frequently reported, with reasonable severity. A similar symptom profile was reported in patients with ATTRwt-CM during a multicenter survey conducted at French referral centers by Damy et al. (40). High proportions of patients reported breathlessness (79%), tingling sensation (∼30%), and difficulty walking (∼30%) prior to diagnosis; 64% of patients felt tired; 49% had disturbed sleep; and 64% felt that their condition limited activities of daily life. Interestingly, whereas previous studies have indicated a detrimental mean delay between first symptoms and diagnosis of up to 6 years (5, 6, 9, 17), our study found a median delay of around 1.5 years, and Damy et al. reported an average 1.6 years in patients with ATTRwt-CM. These findings support additional evaluation of the current diagnostic delays (40).

Our study also included established patient-reported outcome measures, namely the KCCQ, SF-12, HADS, and PROMIS Fatigue and Dyspnea surveys. The KCCQ-OS score has been used to evaluate health status and quality of life in other studies of patients with ATTR-CM, including in the phase III ATTR-ACT (baseline mean of ∼66 for all patients and 65 for those with ATTRwt-CM (15, 41)), in European patients (mean of 65 for all patients and 64 for those with ATTRwt-CM (20)), and in an international survey study (mean of 35 (13)). In a recent longitudinal study, median KCCQ-OS score was 57 in patients with ATTRwt-CM at 12 months after diagnosis and declined by another 5 points by month 36 (17). The median KCCQ-OS score in our study was 68, which is also broadly consistent with expectations for patients with heart failure (42, 43). Comparisons between studies are challenging due to differences in populations such as ATTR-CM severity, clinical characteristics, duration of disease, and therapeutic interventions. However, overall KCCQ data suggest a fair to good health status and quality of life, at best, among patients with ATTR-CM. Our SF-12 findings supported those from the KCCQ, suggesting marginally lower physical and mental health than expected for patients of a similar age (26).

A notable further finding in our study was that patients who were NYHA class III appeared to have a higher burden and potentially a lower quality of life than those who were NYHA class I/II, despite having similar demographics and time since diagnosis. The median KCCQ-OS score in patients who were NYHA class III was 36, which indicates a poor to fair health status (22). This is in keeping with the previously identified correlation between lower KCCQ-OS scores and higher NYHA class in patients with heart failure (22, 44, 45) but lower than recently reported for European patients with ATTR-CM and NYHA class III symptoms (∼55 (20)). The median SF-12 physical component score was 27 in patients who were NYHA class III, which is considerably below the population norms for patients aged ≥75 years (39) (26). This finding particularly highlights the physical burden of advanced ATTR-CM. Another notable finding was that two-thirds (67%) of patients who were NYHA class III had a HADS depression score ≥8, suggesting clinically relevant depression.

### 4.2. Burden in caregivers

Caregivers had a median age of 68 years; the majority were female (85%) and a spouse (59%) or adult child (37%) of the patient. Caregivers lived with patients in two-thirds of pairs, and many reported that patients required help with a range of daily tasks such as cleaning (33%), cooking (27%), bathing (18%), and walking (18%). Around 10% of caregivers reported that their caregiving responsibilities meant that they could not complete their typical daily chores at least once in the prior 3 months.

In an unpaired evaluation of burden in caregivers to patients with ATTR amyloidosis and a cardiac, neurological, or mixed phenotype by Stewart et al., caregivers had a mean age of 56 years, 69% were female, and 72% were the spouse of the patient (13). A high proportion of caregivers had transthyretin amyloidosis themselves (41% vs. 0% in our study) and they had provided care for 3 (in the US) to 7 years (in Spain, vs. 1.5 years in our study), currently spending an average of 46 h per week caring for a patient with ATTR amyloidosis (vs. 4.5 h in our study). Mean ZBI score in that analysis was 34 among caregivers from the US, and 13 among those from Spain; and this compares with a median of 13 in our study. We found that just over one-third of caregivers (35%) experienced at least a mild to moderate burden of care using the ZBI (score ≥21). Both Stewart et al. and a qualitative study have suggested notable depression and anxiety among caregivers for patients with ATTR-CM, but this was not reflected in our findings (9, 13). Previous data on caregiver burden are very limited but may indicate a higher burden of care than in our study. This is likely to reflect several inconsistencies in the patient and caregiver populations studied, including a longer duration of care and societal, disease severity, and demographic differences.

Caregivers in our study reported a numerically higher burden when caring for patients with more severe heart failure. Over half (51%) of caregivers to patients who were NYHA class III reported at least a mild to moderate burden of care, as compared with one-third (33%) of those to patients who were NYHA class I/II. High proportions of caregivers to patients who were NYHA class III reported that the patient required help with daily activities including cleaning (58%), cooking (51%), bathing (35%), and walking (35%). We did not find that caregivers' health status, anxiety, depression, or fatigue symptoms were related to the severity of the patients' heart failure. However, this study comprised caregivers who had been providing care for an average of 1.5 years to patients who were newly diagnosed and, as yet, untreated with disease-modifying therapy. The character and magnitude of caregiver burden might differ over the course of prolonged caregiving.

### 4.3. Limitations

Limitations of our study include the potential for self-reporting bias. Equally, data provided by the investigator might differ from those elicited by asking the same question to the patient. There is also potential selection bias, as enrolled patient and caregiver pairs were from centers of excellence or referral centers, and their experience may not be reflective of those managed outside these centers. Further, while the aim of the study was to evaluate burden in patients who were not treated with disease-modifying therapy, this represents a subpopulation, and the reasons for lack of treatment were not captured. This study was conducted during the COVID-19 pandemic, meaning that data on healthcare utilization may not be representative of usual clinical practice. As mentioned above, interpretation of our data should be limited to the population of our survey. The specific nature of this population also makes comparisons with existing studies in ATTR-CM or other diseases complicated, as population and disease characteristics can have significant effects on burden. Lastly, our interpretation of the data is based on the observed descriptive differences. Further analyses comparing groups using statistical modeling would be necessary to confirm our conclusions and should take into account the numerous factors that can influence the outcomes of each burden measure.

## 5. Conclusion

This was the first multicenter, international, real-world study to comprehensively characterize the burden of ATTR-CM in patients naïve to disease-modifying treatment and their caregivers. Findings suggested that ATTR-CM was a burden to both patients and caregivers, despite these populations being relatively newly diagnosed and with a short caregiving duration, respectively.

## Data Availability

Upon request, and subject to review, Pfizer will provide the data that support the findings of this study. Subject to certain criteria, conditions, and exceptions, Pfizer may also provide access to the related individual de-identified participant data. See https://www.pfizer.com/science/clinical-trials/trial-data-and-results for more information. Please direct any further enquiries to the corresponding author.
