# Supplementary material for: Burden of untreated transthyretin amyloid cardiomyopathy on patients and their caregivers by disease severity: results from a multicenter, non-interventional, real-world study
Source: Front Cardiovasc Med. 2023 Aug 29;10:1238843. doi: 10.3389/fcvm.2023.1238843 (PMC10497948; doi:10.3389/fcvm.2023.1238843)
Supplement: Supplementary file 1 [file Datasheet1.pdf]

Plain Language Summary

Please note that this summary only contains information from the full scientific article:

View Scientific Article

Burden of untreated transthyretin amyloid cardiomyopathy on people living with the condition and their caregivers

Date of summary: July 2023

Study start date: July 2021

Study end date: August 2022

The full title of this article: Burden of untreated transthyretin amyloid cardiomyopathy on patients and their caregivers by disease severity: results from a multicenter, non-interventional, real-world study

Key takeaways

- In this study, researchers used questionnaires to ask people with **transthyretin amyloid cardiomyopathy** (**ATTR-CM** for short) about the burden of living with the condition.
  - The study specifically included people with ATTR-CM who were not receiving treatment that corrected the cause of their condition.
  - Researchers found that people with ATTR-CM had a range of symptoms. These symptoms impacted on many parts of their lives.
  - Symptoms reduced their ability to walk, look after themselves, and take part in daily activities. They also made them feel worse about their health. In these ways, ATTR-CM is a burden.
- Researchers also asked caregivers of the people with ATTR-CM about how the condition is a burden to them.
  - They found that caregivers provided help with a range of physical tasks. This sometimes stopped them from completing their own daily activities. In these ways, ATTR-CM is a burden.
- The burden of ATTR-CM was higher on people with the condition and their caregivers when the person with ATTR-CM had more severe symptoms of heart failure.

Amyloid
<A-muh-loyd>

Cardiomyopathy
<KAR-dee-oh-my-OP-uh-thee>

Transthyretin
<trans-thy-REH-tin>

Glossary

Amyloid: clumps or strands of mis-shaped proteins

Anxiety: feelings of worry and stress

Cardiomyopathies: conditions that affect heart muscle

Depression: long-lasting feelings of unhappiness or hopelessness

Dyspnea: shortness of breath

Fatigue: mental and physical tiredness

Gene: a segment of DNA that tells the body how to make a protein

Heart failure: when the heart can't pump enough blood around the body

Transthyretin: a protein mainly made by the liver

Additional information

More information can be found in the scientific article of this study, which you can access here:

View Scientific Article

Amyloid
<A-muh-loyd>

Cardiomyopathy
<KAR-dee-oh-my-OP-uh-thee>

Transthyretin
<trans-thy-REH-tin>

Glossary

Amyloid: clumps or strands of mis-shaped proteins

Anxiety: feelings of worry and stress

Cardiomyopathies: conditions that affect heart muscle

Depression: long-lasting feelings of unhappiness or hopelessness

Dyspnea: shortness of breath

Fatigue: mental and physical tiredness

Gene: a segment of DNA that tells the body how to make a protein

Heart failure: when the heart can't pump enough blood around the body

Transthyretin: a protein mainly made by the liver

Additional information

More information can be found in the scientific article of this study, which you can access here:

View Scientific Article

What did this study look at?

What is ATTR-CM?

- **Cardiomyopathies** are conditions that affect heart muscle.
- **ATTR-CM** is a type of cardiomyopathy that becomes worse over time (also called a progressive disease).

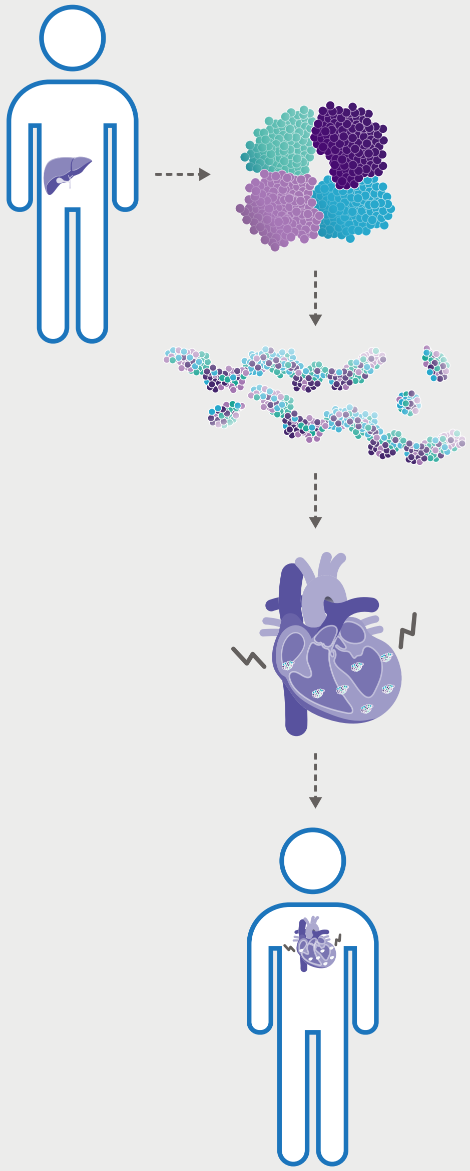

Transthyretin is a protein mainly made by the liver. It circulates around the body in the blood.

Transthyretin amyloid is formed when transthyretin protein changes shape and forms clumps and strands.

Transthyretin amyloid can build up in the heart, making the walls thick and stiff. This causes cardiomyopathy and stops the heart from pumping properly.

Amyloid build up over time can lead to heart failure, where the heart can't pump enough blood around the body.

Transthyretin amyloid can also build up in other areas of the body and cause problems unrelated to the heart, such as muscle weakness, numbness, tingling, or pain.

- There are two types of ATTR-CM:
  1. The most common type occurs with aging and is mostly diagnosed in men around 70 to 80 years old. This is called **wild-type ATTR-CM**.
  2. The other type of ATTR-CM is caused by the transthyretin gene passed to a person from their parent. This is called **variant ATTR-CM**.
     - A gene is a segment of DNA that tells the body how to make a protein.
     - Some people have a gene for a transthyretin protein that is more likely to form amyloid.
     - Variant ATTR-CM can develop at an earlier age than wild-type ATTR-CM.
     - People with variant ATTR-CM more commonly have symptoms in other areas of the body than people with wild-type ATTR-CM.

Amyloid
<A-muh-loyd>

Cardiomyopathy
<KAR-dee-oh-my-OP-uh-thee>

Transthyretin
<trans-thy-REH-tin>

What was this study about?

- This study **asked people with ATTR-CM and their main caregiver about the ways in which the condition is a burden** to them.
  - Burden is a way to describe how a condition affects a person.
  - Burden has many aspects and means different things to different people.
  - Learning about burden can help researchers understand how people with ATTR-CM and their caregivers feel about the challenges they face.
- Researchers asked people with ATTR-CM and their main caregiver about their daily life and how they felt about their health.
  - The study combined several **questionnaires** to look at a different aspects of burden. Many have been used in other studies.
  - Questionnaires that ask people how they feel about their health are sometimes called patient-reported outcome surveys.
- This was the first study to ask people with ATTR-CM and their caregivers, from **many countries and continents**, about their burden.
  - It specifically included people who were **not receiving treatment** that corrected the cause of their condition.
  - Findings from **people with ATTR-CM and their main caregiver were collected separately and then paired**.
- There are many ways to measure the severity of cardiomyopathies such as ATTR-CM.
  - This study used a scale called the New York Heart Association (or NYHA for short) Functional Classification to measure **how severe each person's heart failure symptoms** were.
  - Researchers grouped people with ATTR-CM by the severity of their heart failure symptoms and looked at the burden felt by them and their caregivers.

NYHA classification by heart failure symptoms

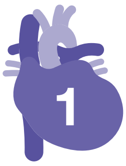

Limited/no heart failure symptoms

People can do normal physical activities, like walking, without symptoms. People are not physically limited.

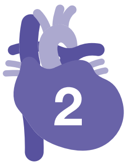

Mild heart failure symptoms

People have symptoms such as tiredness or shortness of breath when doing normal physical activities.

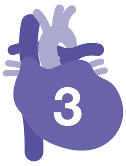

Moderate heart failure symptoms

People have symptoms such as tiredness or shortness of breath that limit their normal physical activities.

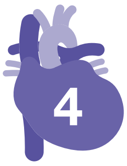

Severe heart failure symptoms

People have symptoms such as tiredness or shortness of breath when resting. People are largely unable to do normal physical activities.

Glossary

Amyloid: clumps or strands of mis-shaped proteins

Anxiety: feelings of worry and stress

Cardiomyopathies: conditions that affect heart muscle

Depression: long-lasting feelings of unhappiness or hopelessness

Dyspnea: shortness of breath

Fatigue: mental and physical tiredness

Gene: a segment of DNA that tells the body how to make a protein

Heart failure: when the heart can't pump enough blood around the body

Transthyretin: a protein mainly made by the liver

Additional information

More information can be found in the scientific article of this study, which you can access here:

View Scientific Article

Amyloid
<A-muh-loyd>

Cardiomyopathy
<KAR-dee-oh-my-OP-uh-thee>

Transthyretin
<trans-thy-REH-tin>

Glossary

Amyloid: clumps or strands of mis-shaped proteins

Anxiety: feelings of worry and stress

Cardiomyopathies: conditions that affect heart muscle

Depression: long-lasting feelings of unhappiness or hopelessness

Dyspnea: shortness of breath

Fatigue: mental and physical tiredness

Gene: a segment of DNA that tells the body how to make a protein

Heart failure: when the heart can't pump enough blood around the body

Transthyretin: a protein mainly made by the liver

Additional information

More information can be found in the scientific article of this study, which you can access here:

View Scientific Article

Who took part in this study?

208 pairs of people with ATTR-CM and their main unpaid caregiver completed questionnaires between July 2021 and August 2022

208 people with ATTR-CM

208 caregivers

For each pair, additional information was provided by the person with ATTR-CM's doctor

Location of the 208 pairs:

- 95 from Italy
- 31 from Spain
- 34 from France
- 17 from Australia
- 10 from Canada
- 15 from Austria
- 6 from Russia

- Not all questions were fully completed. Some information was missing, including:
  - The type of ATTR-CM was missing for 53 people with ATTR-CM (1 in 4).
  - The severity of heart failure symptoms was missing for 9 people with ATTR-CM (4 in 100).

Amyloid
<A-muh-loyd>

Cardiomyopathy
<KAR-dee-oh-my-OP-uh-thee>

Transthyretin
<trans-thy-REH-tin>

Characteristics of people with ATTR-CM

Average **age**: **81** years

Age range: **46 to 90** years

Average **time since diagnosis**: **6** months

9 in 10 people were **men**

1 in 10 people were **women**

9 in 10 people had **wild-type ATTR-CM**

1 in 10 people had **variant ATTR-CM**

8 in 10 people had **mild or no** heart failure symptoms

2 in 10 people had **moderate** heart failure symptoms

1 in 10 people had diagnosed **depression**

1 in 10 people had diagnosed **anxiety**

Glossary

Amyloid: clumps or strands of mis-shaped proteins

Anxiety: feelings of worry and stress

Cardiomyopathies: conditions that affect heart muscle

Depression: long-lasting feelings of unhappiness or hopelessness

Dyspnea: shortness of breath

Fatigue: mental and physical tiredness

Gene: a segment of DNA that tells the body how to make a protein

Heart failure: when the heart can't pump enough blood around the body

Transthyretin: a protein mainly made by the liver

Additional information

More information can be found in the scientific article of this study, which you can access here:

View Scientific Article

Amyloid
<A-muh-loyd>

Cardiomyopathy
<KAR-dee-oh-my-OP-uh-thee>

Transthyretin
<trans-thy-REH-tin>

- People with ATTR-CM were **most often men aged more than 80 years old with newly diagnosed wild-type ATTR-CM**.
  - **None received treatment** that corrected the cause of their condition.
  - **Most had mild or no heart failure symptoms** (NYHA class 1 or 2). Some had moderate heart failure symptoms (class 3), and none had severe heart failure symptoms (class 4).
  - Age, gender, time since diagnosis, and the type of ATTR-CM were generally similar in people with mild or no heart failure symptoms and people with moderate heart failure symptoms.

Characteristics of caregivers

Average **age**: 68 years

Age range: 32 to 88 years

Average **duration of caregiving** was 1½ years

Average **time spent providing care each week** was 4½ hours

8 in 10 caregivers were **women**

2 in 10 caregivers were **men**

6 in 10 caregivers were **married or in a domestic partnership** with the person who had ATTR-CM

4 in 10 caregivers were **the son or daughter** of the person with ATTR-CM

7 in 10 caregivers **lived with** the person with ATTR-CM

Glossary

Amyloid: clumps or strands of mis-shaped proteins

Anxiety: feelings of worry and stress

Cardiomyopathies: conditions that affect heart muscle

Depression: long-lasting feelings of unhappiness or hopelessness

Dyspnea: shortness of breath

Fatigue: mental and physical tiredness

Gene: a segment of DNA that tells the body how to make a protein

Heart failure: when the heart can't pump enough blood around the body

Transthyretin: a protein mainly made by the liver

Additional information

More information can be found in the scientific article of this study, which you can access here:

View Scientific Article

- Caregivers who took part in the study were **most often female partners or adult daughters of the person with ATTR-CM**. They usually lived with the person they cared for.
- On average, caregivers to people with moderate heart failure symptoms had been providing care for longer than caregivers to people with mild or no heart failure symptoms (2 years compared to 1 year).
  – They also spent more hours per week providing care (average of 17½ hours compared to 2 hours).
- Most other characteristics were similar in caregivers to people with different severities of heart failure symptoms.

What were the results of the study?

Burden reported by people with ATTR-CM

Symptoms

- People with ATTR-CM took medicines to lessen their symptoms, as was recommended in their country and by their doctor. They did not receive treatment that corrected the cause of their condition.
- **Current symptoms in people with ATTR-CM** were reported by their doctor from a pre-defined list. All of the symptoms are burdens.
  – People with ATTR-CM had symptoms that **affected many areas** of their body.
  – All of the most common symptoms were more often reported in people with moderate heart failure symptoms than in people with mild or no heart failure symptoms.

Most common symptoms of ATTR-CM in people with symptoms of moderate heart failure and mild or no heart failure.

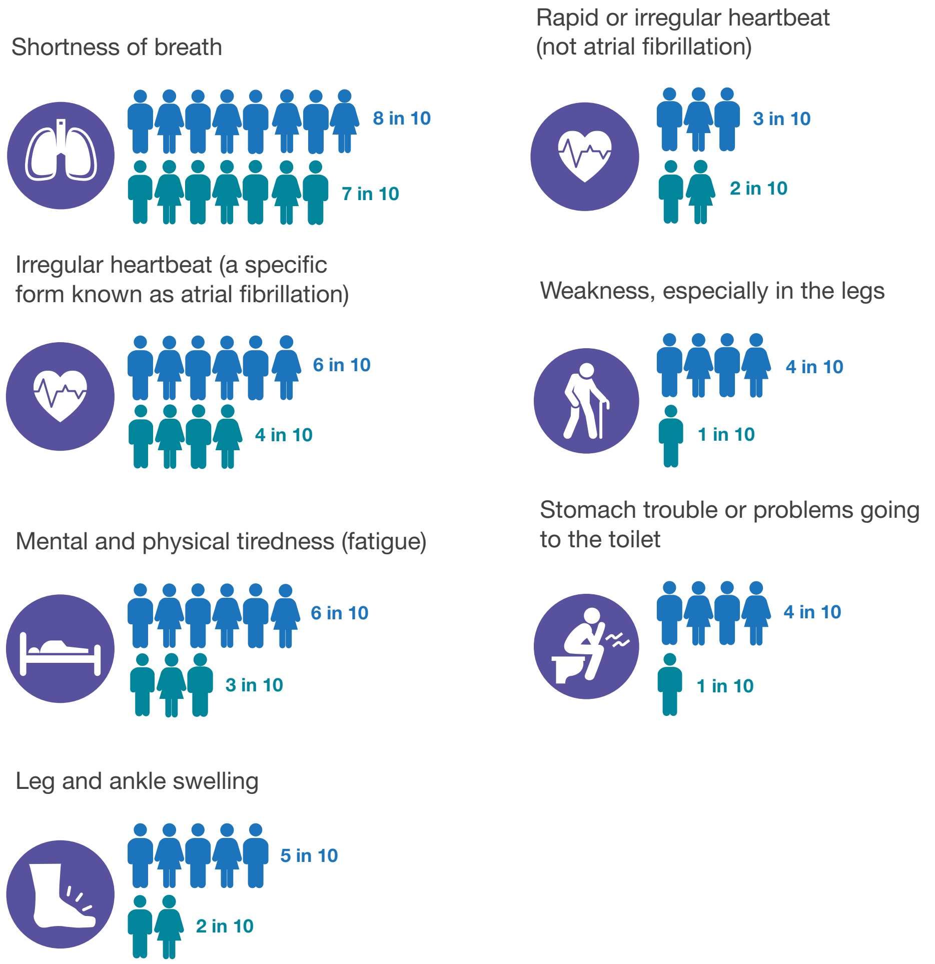

Amyloid
<A-muh-loyd>

Cardiomyopathy
<KAR-dee-oh-my-OP-uh-thee>

Transthyretin
<trans-thy-REH-tin>

Glossary

Amyloid: clumps or strands of mis-shaped proteins

Anxiety: feelings of worry and stress

Cardiomyopathies: conditions that affect heart muscle

Depression: long-lasting feelings of unhappiness or hopelessness

Dyspnea: shortness of breath

Fatigue: mental and physical tiredness

Gene: a segment of DNA that tells the body how to make a protein

Heart failure: when the heart can't pump enough blood around the body

Transthyretin: a protein mainly made by the liver

Additional information

More information can be found in the scientific article of this study, which you can access here:

View Scientific Article

Amyloid
<A-muh-loyd>

Cardiomyopathy
<KAR-dee-oh-my-OP-uh-thee>

Transthyretin
<trans-thy-REH-tin>

- People with ATTR-CM often reported that they had **problems walking**. Many people had also recently been **unable to take part in social, leisure, or household activities**.
  - These reports were more common in people with moderate heart failure symptoms than with mild or no heart failure symptoms.

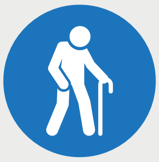

Proportion of people who could not walk normally due to the symptoms of ATTR-CM

8 in 10

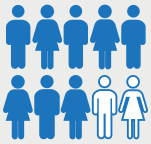

people with **moderate heart failure** symptoms

6 in 10

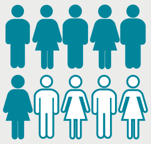

people with **mild or no heart failure** symptoms

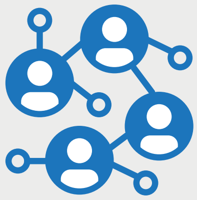

Proportion of people who reported times they had been unable to take part in social or leisure activities in the last 3 months

5 in 10

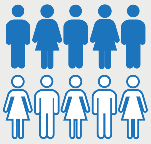

people with **moderate heart failure** symptoms

2 in 10

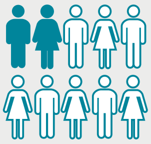

people with **mild or no heart failure** symptoms

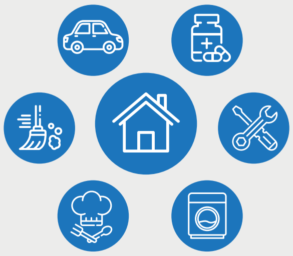

Proportion of people who reported times they had been unable to do household chores in the last 3 months

5 in 10

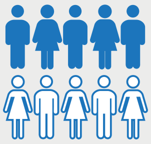

people with **moderate heart failure** symptoms

2 in 10

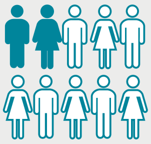

people with **mild or no heart failure** symptoms

Glossary

Amyloid: clumps or strands of mis-shaped proteins

Anxiety: feelings of worry and stress

Cardiomyopathies: conditions that affect heart muscle

Depression: long-lasting feelings of unhappiness or hopelessness

Dyspnea: shortness of breath

Fatigue: mental and physical tiredness

Gene: a segment of DNA that tells the body how to make a protein

Heart failure: when the heart can't pump enough blood around the body

Transthyretin: a protein mainly made by the liver

Additional information

More information can be found in the scientific article of this study, which you can access here:

View Scientific Article

Amyloid
<A-muh-loyd>

Cardiomyopathy
<KAR-dee-oh-my-OP-uh-thee>

Transthyretin
<trans-thy-REH-tin>

Kansas City Cardiomyopathy Questionnaire Overall Summary score

- The Kansas City Cardiomyopathy Questionnaire Overall Summary score (or **KCCQ-OS score** for short) measures **how a person feels their health was** over the last 2 weeks.
  - The final score can range from 0 to 100. A lower KCCQ-OS score suggests that a person has a lower quality of life, more symptoms, or that the condition is more of a burden on them.
- People with ATTR-CM had an **average KCCQ-OS score of 68**. Using standard scoring, this suggests that they **felt that their health was fair to good**.
  - The average KCCQ-OS score was lower in people with moderate heart failure symptoms than in people with mild or no heart failure symptoms. This suggests people with moderate symptoms felt worse about their health.

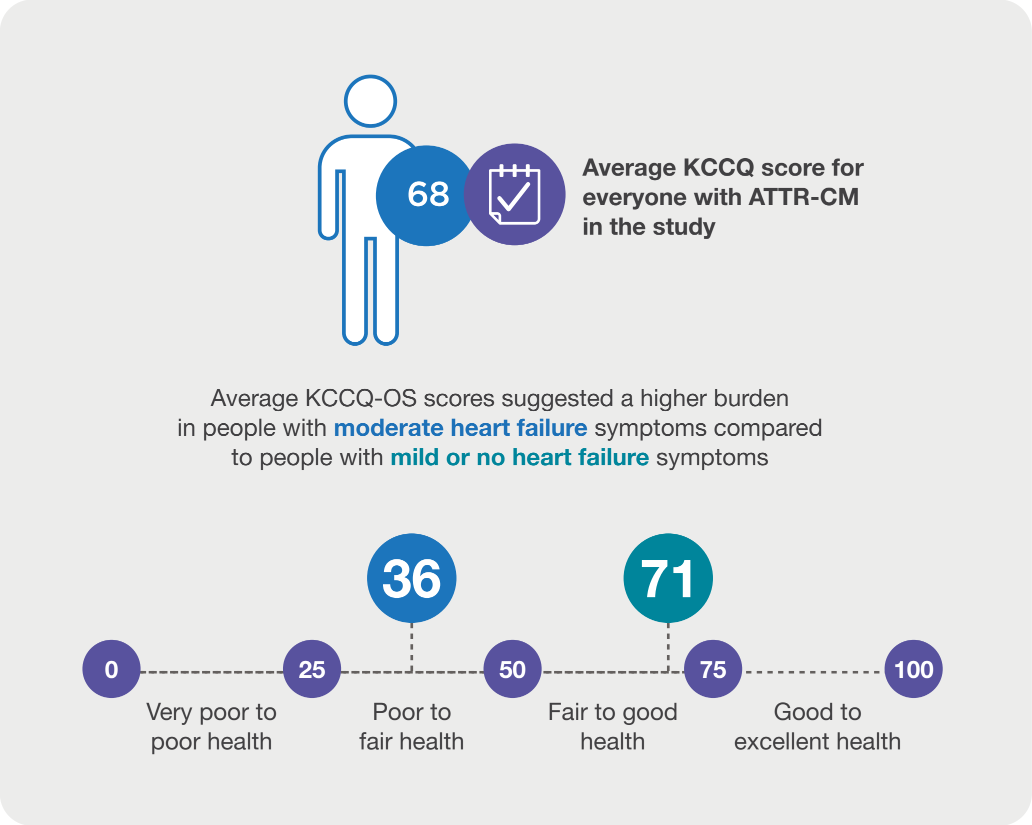

Glossary

Amyloid: clumps or strands of mis-shaped proteins

Anxiety: feelings of worry and stress

Cardiomyopathies: conditions that affect heart muscle

Depression: long-lasting feelings of unhappiness or hopelessness

Dyspnea: shortness of breath

Fatigue: mental and physical tiredness

Gene: a segment of DNA that tells the body how to make a protein

Heart failure: when the heart can't pump enough blood around the body

Transthyretin: a protein mainly made by the liver

Additional information

More information can be found in the scientific article of this study, which you can access here:

View Scientific Article

The 12-item Short Form Health Survey score

- The 12-item Short Form Health Survey (or **SF-12** for short) is used to measure **how a person feels their health was** over the last 7 days.
  - It provides two scores. One suggests how a person feels about their mental health, and the other suggests how they feel about their physical health.
  - Both parts have a score that ranges from 0 to 100. A lower SF-12 score suggests that a person feels worse about their health.
  - People of different ages are expected to have different SF-12 scores. For example, older people are expected to have lower scores for physical health than younger people.
- People with ATTR-CM had an average **SF-12 mental health score of 47**. This is just below the average for people aged 75 years or more (score of 54). This suggests that they had **slightly poorer mental health than people of a similar age** in the general population.
  - SF-12 mental health scores were lower in people with moderate heart failure symptoms than in people with mild or no heart failure symptoms. This suggests people with moderate symptoms felt worse about their mental health.

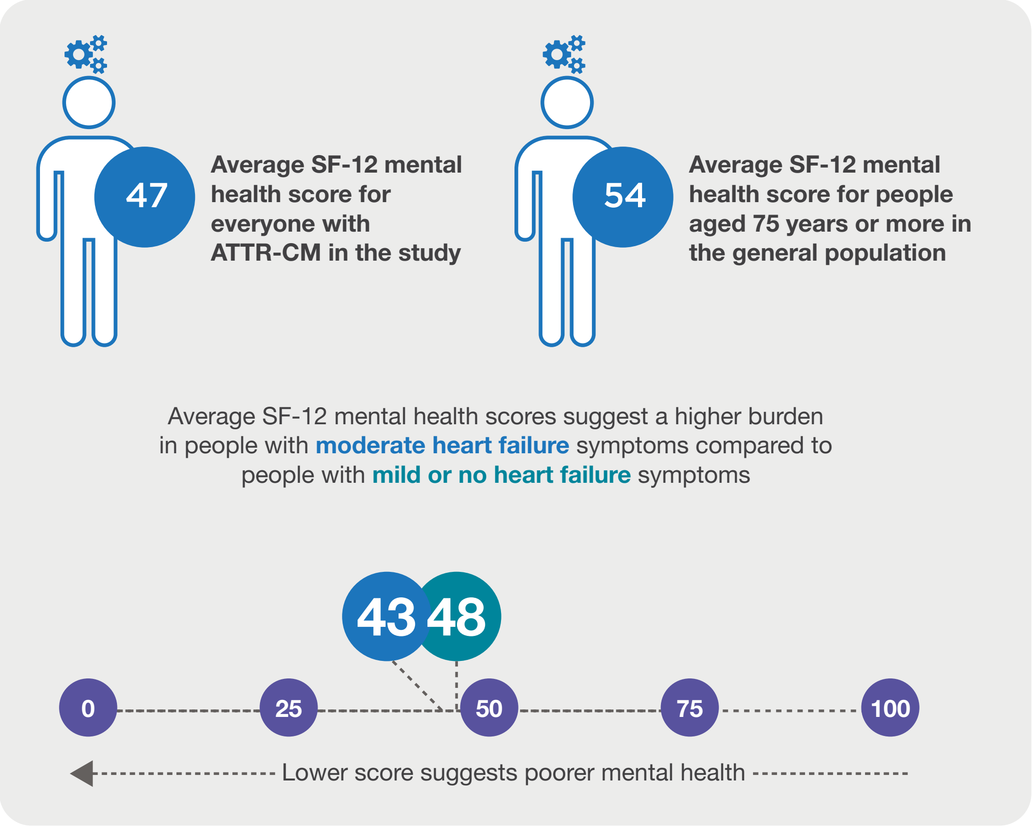

Amyloid
<A-muh-loyd>

Cardiomyopathy
<KAR-dee-oh-my-OP-uh-thee>

Transthyretin
<trans-thy-REH-tin>

Glossary

Amyloid: clumps or strands of mis-shaped proteins

Anxiety: feelings of worry and stress

Cardiomyopathies: conditions that affect heart muscle

Depression: long-lasting feelings of unhappiness or hopelessness

Dyspnea: shortness of breath

Fatigue: mental and physical tiredness

Gene: a segment of DNA that tells the body how to make a protein

Heart failure: when the heart can't pump enough blood around the body

Transthyretin: a protein mainly made by the liver

Additional information

More information can be found in the scientific article of this study, which you can access here:

View Scientific Article

Amyloid
<A-muh-loyd>

Cardiomyopathy
<KAR-dee-oh-my-OP-uh-thee>

Transthyretin
<trans-thy-REH-tin>

- People with ATTR-CM had an average **SF-12 physical health score of 36**. This is just below the average for people aged 75 years or more (score of 39). This suggests that they had **slightly poorer physical health than people of a similar age** in the general population.
  – SF-12 physical health scores were lower in people with moderate heart failure symptoms than in people with mild or no heart failure symptoms. This suggests people with moderate symptoms felt their physical health was worse.

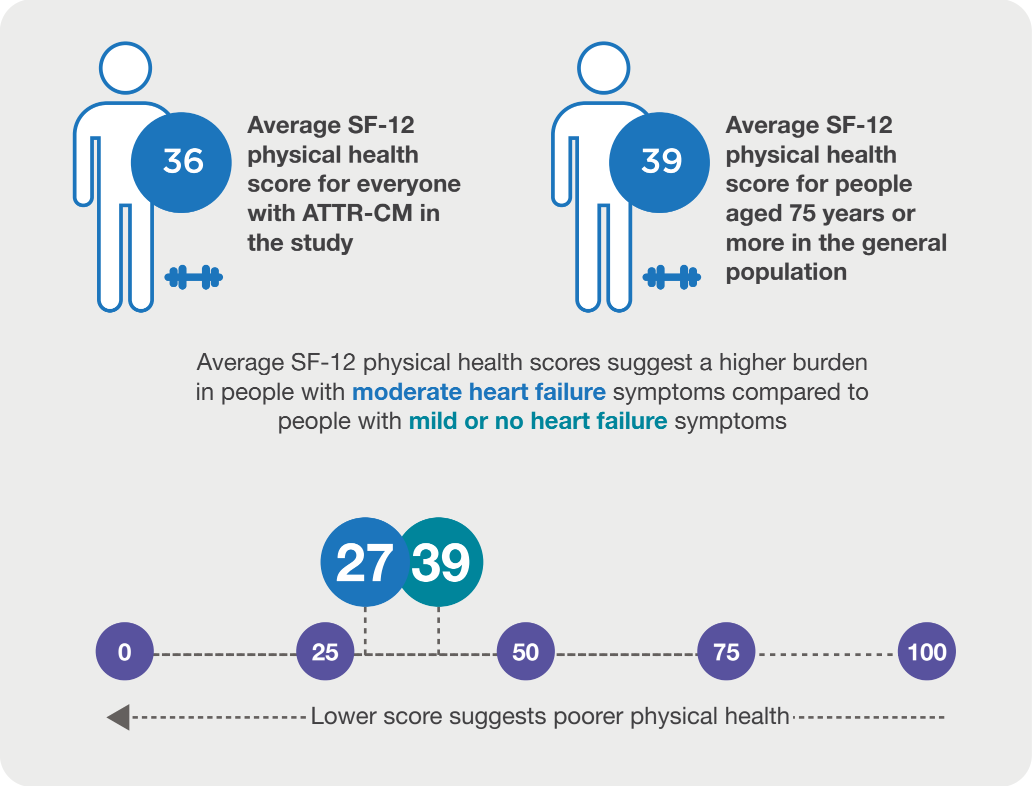

Hospital Anxiety and Depression Scale

- The Hospital Anxiety and Depression Scale (or **HADS** for short) is used to look for signs of **anxiety and depression** in a person.
  – The final score has two parts. One can be used to indicate possible anxiety, and another can be used to indicate possible depression.
  – Each part has a score ranging from 0 to 21. A score of 8 or more in either part suggests that a person might have anxiety or depression. This may or may not be diagnosed already.
- Findings from the HADS suggested that **depression was more common** among people with moderate heart failure symptoms than people with mild or no heart failure symptoms.
  – Most people with ATTR-CM did not report scores that suggested that they might have **anxiety**.

Findings from the HADs survey suggested that….

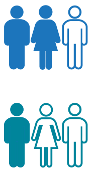

2 in 3 people with **moderate heart failure** symptoms had depression

1 in 3 people with **mild or no heart failure** symptoms had depression

Glossary

Amyloid: clumps or strands of mis-shaped proteins

Anxiety: feelings of worry and stress

Cardiomyopathies: conditions that affect heart muscle

Depression: long-lasting feelings of unhappiness or hopelessness

Dyspnea: shortness of breath

Fatigue: mental and physical tiredness

Gene: a segment of DNA that tells the body how to make a protein

Heart failure: when the heart can't pump enough blood around the body

Transthyretin: a protein mainly made by the liver

Additional information

More information can be found in the scientific article of this study, which you can access here:

View Scientific Article

PROMIS scales

- Patient-Reported Outcomes Measurement Information System scales, or **PROMIS scales** for short, are used to ask people about the **severity of a symptom** over the last 7 days.
  - PROMIS scales for fatigue and dyspnea were included in this study because they are common symptoms in people with ATTR-CM.
  - **Fatigue** is a medical term for mental and physical tiredness.
  - **Dyspnea** is a medical term for shortness of breath.
  - In these scales, a higher score suggests that a person has more severe fatigue or dyspnea.
- People with ATTR-CM had an average **PROMIS Fatigue** score of 51. This suggests that they had **similar mental and physical tiredness to people in the general population (average score of 50)**.
  - The PROMIS Fatigue score was slightly higher in people with moderate heart failure symptoms (score 57) than in people with mild or no heart failure symptoms (score 50).
- Less than half of people with ATTR-CM completed the **PROMIS Dyspnea** survey. The final score is usually compared to that from people with a serious lung condition called chronic obstructive pulmonary disease (or COPD for short).
  - In COPD, the lungs are inflamed and damaged. This can cause breathing problems, including shortness of breath.
- People with ATTR-CM had an average PROMIS Dyspnea score of 43, which is less than people with COPD (score of 50). This suggests that they had **less shortness of breath than people with COPD**.
  - The PROMIS Dyspnea score was slightly higher in people with moderate heart failure symptoms (score 48) than in people with mild or no heart failure symptoms (score 42).

Amyloid
<A-muh-loyd>

Cardiomyopathy
<KAR-dee-oh-my-OP-uh-thee>

Transthyretin
<trans-thy-REH-tin>

Glossary

Amyloid: clumps or strands of mis-shaped proteins

Anxiety: feelings of worry and stress

Cardiomyopathies: conditions that affect heart muscle

Depression: long-lasting feelings of unhappiness or hopelessness

Dyspnea: shortness of breath

Fatigue: mental and physical tiredness

Gene: a segment of DNA that tells the body how to make a protein

Heart failure: when the heart can't pump enough blood around the body

Transthyretin: a protein mainly made by the liver

Additional information

More information can be found in the scientific article of this study, which you can access here:

View Scientific Article

Amyloid
<A-muh-loyd>

Cardiomyopathy
<KAR-dee-oh-my-OP-uh-thee>

Transthyretin
<trans-thy-REH-tin>

Glossary

Amyloid: clumps or strands of mis-shaped proteins

Anxiety: feelings of worry and stress

Cardiomyopathies: conditions that affect heart muscle

Depression: long-lasting feelings of unhappiness or hopelessness

Dyspnea: shortness of breath

Fatigue: mental and physical tiredness

Gene: a segment of DNA that tells the body how to make a protein

Heart failure: when the heart can't pump enough blood around the body

Transthyretin: a protein mainly made by the liver

Additional information

More information can be found in the scientific article of this study, which you can access here:

View Scientific Article

Burden of ATTR-CM on caregivers

- Caregivers to people with ATTR-CM completed the same HADS, SF-12, and PROMIS Fatigue surveys as previously described.
- They were also asked about the physical tasks that patients required help with, and about how much overall burden they felt.

Physical tasks that the person requires help with

- Many caregivers said that the person that they cared for **needed help with some common daily activities**.
  - Needing help was more common in people with moderate heart failure symptoms than with mild or no heart failure symptoms.

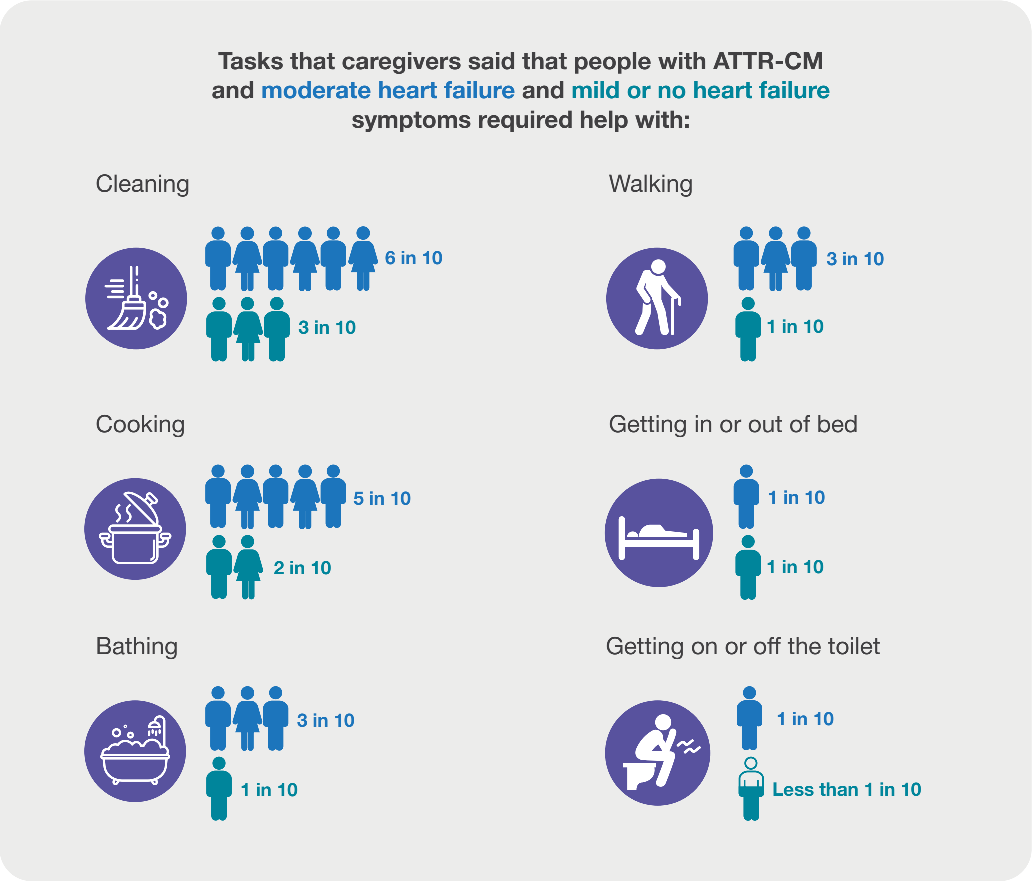

- Some caregivers to people with ATTR-CM **could not complete their own daily chores because of their caregiving responsibilities**.
  - This was more common in caregivers to people with moderate heart failure symptoms than with mild or no heart failure symptoms.

In the last 3 months, ...

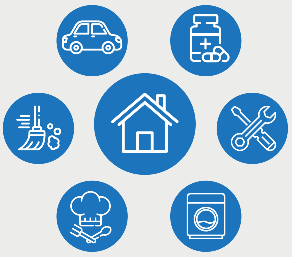

the proportion of caregivers who reported times that they had been unable to complete their own household chores due to caregiving responsibilities was:

1 in 10

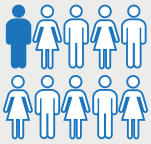

for caregivers to people with ATTR-CM and **moderate heart failure**

1 in 10

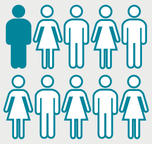

caregivers to people ATTR-CM and **mild or no heart failure** symptoms

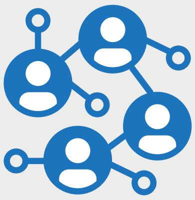

the proportion of caregivers who reported times that they needed help from a family member to do their own household chores was:

2 in 10

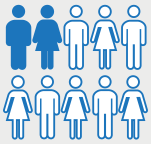

for caregivers to people with ATTR-CM and **moderate heart failure** symptoms

1 in 10

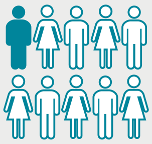

for caregivers to people ATTR-CM and **mild or no heart failure** symptoms

Amyloid
<A-muh-loyd>

Cardiomyopathy
<KAR-dee-oh-my-OP-uh-thee>

Transthyretin
<trans-thy-REH-tin>

Glossary

Amyloid: clumps or strands of mis-shaped proteins

Anxiety: feelings of worry and stress

Cardiomyopathies: conditions that affect heart muscle

Depression: long-lasting feelings of unhappiness or hopelessness

Dyspnea: shortness of breath

Fatigue: mental and physical tiredness

Gene: a segment of DNA that tells the body how to make a protein

Heart failure: when the heart can't pump enough blood around the body

Transthyretin: a protein mainly made by the liver

Additional information

More information can be found in the scientific article of this study, which you can access here:

View Scientific Article

Amyloid
<A-muh-loyd>

Cardiomyopathy
<KAR-dee-oh-my-OP-uh-thee>

Transthyretin
<trans-thy-REH-tin>

Zarit Burden Interview score

- The Zarit Burden Interview (or **ZBI** for short) is used to **measure the burden felt by caregivers**.
  - The final score can range from 0 to 88. A higher score suggests that a caregiver feels more burden.
- 4 in 10 caregivers to people with ATTR-CM had a ZBI score between 21 and 61. This score suggests that they felt at least a **mild to moderate burden** of care.

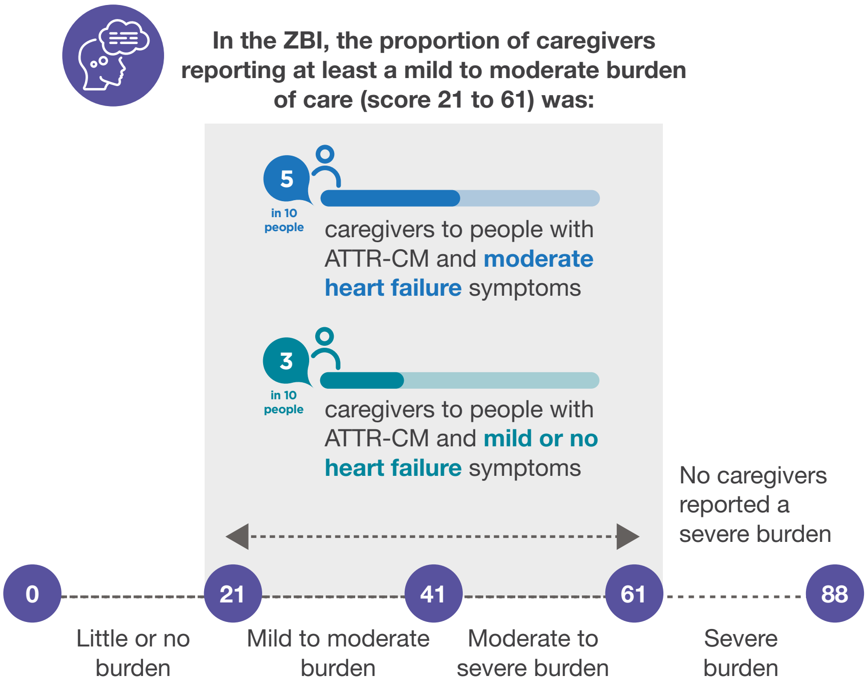

SF-12, HADs, and PROMIS scores

- Caregivers to people with moderate heart failure symptoms and caregivers to people with mild or no heart failure symptoms reported **similar scores from the SF-12, HADs, and PROMIS Fatigue surveys**.

More results from this study can be found here: **View Scientific Article**

Glossary

Amyloid: clumps or strands of mis-shaped proteins

Anxiety: feelings of worry and stress

Cardiomyopathies: conditions that affect heart muscle

Depression: long-lasting feelings of unhappiness or hopelessness

Dyspnea: shortness of breath

Fatigue: mental and physical tiredness

Gene: a segment of DNA that tells the body how to make a protein

Heart failure: when the heart can't pump enough blood around the body

Transthyretin: a protein mainly made by the liver

Additional information

More information can be found in the scientific article of this study, which you can access here:

View Scientific Article

What were the main conclusions reported by the researchers?

- Researchers found that both **people with ATTR-CM and their caregivers felt the condition was a burden to them**.
  - In both groups, the burden appeared to be worse when the person with ATTR-CM had more severe heart failure symptoms.
- **People with ATTR-CM had a wide range of symptoms** that sometimes stopped them from doing usual daily tasks. This affected their quality of life and some people had signs of depression. Many people with ATTR-CM didn't feel as good about their health as was expected for people their age.
- **Caregivers to people with ATTR-CM helped with a range of daily tasks, often at the expense of their own activities**.
- In this study, people with ATTR-CM mostly had the wild-type form. The burden reported in this study might not be the same as in people with other conditions caused by amyloid build up.

Who sponsored this study?

This study was sponsored by Pfizer.

Pfizer Inc.
235 East 42nd Street NY, NY 10017
Phone (United States): +1 212-733-2323

Pfizer thanks all of the people who took part in this study.

Scientific Article
Lucia Ponti, Kristen Hsu, Thibaud Damy, Eduardo Villacorta, Nicolas Verheyen, Denis Keohane, Ronnie Wang, Monica Ines, Nisith Kumar, Carmen Munteanu, and Francesco Cappelli. Burden of untreated transthyretin amyloid cardiomyopathy on patients and their caregivers by disease severity: results from a multicenter, non-interventional, real-world study. Front Cardiovasc Med. 10:1238843. doi: 10.3389/fcvm.2023.1238843

Acknowledgments
Writing support for this summary was provided by Jennifer Bodkin at Engage Scientific Solutions and was funded by Pfizer.

Authors of the full article were involved in preparing this summary.

The purpose of this plain language summary is to help you to understand the findings from recent research.

- This summary reports the results of a single study. The results of this study may differ from those of other studies. Health professionals should make treatment decisions based on all available evidence, not on the results of a single study.

Amyloid
<A-muh-loyd>

Cardiomyopathy
<KAR-dee-oh-my-OP-uh-thee>

Transthyretin
<trans-thy-REH-tin>

Glossary

Amyloid: clumps or strands of mis-shaped proteins

Anxiety: feelings of worry and stress

Cardiomyopathies: conditions that affect heart muscle

Depression: long-lasting feelings of unhappiness or hopelessness

Dyspnea: shortness of breath

Fatigue: mental and physical tiredness

Gene: a segment of DNA that tells the body how to make a protein

Heart failure: when the heart can't pump enough blood around the body

Transthyretin: a protein mainly made by the liver

Additional information

More information can be found in the scientific article of this study, which you can access here:

View Scientific Article
